# Direct Assessment of the Effect of the Gly380Arg Achondroplasia Mutation on FGFR3 Dimerization Using Quantitative Imaging FRET

**DOI:** 10.1371/journal.pone.0046678

**Published:** 2012-10-09

**Authors:** Jesse Placone, Kalina Hristova

**Affiliations:** Department of Materials Science and Engineering, Johns Hopkins University, Baltimore, Maryland, United States of America; Baylor College of Medicine, United States of America

## Abstract

The Gly380Arg mutation in FGFR3 is the genetic cause for achondroplasia (ACH), the most common form of human dwarfism. The mutation has been proposed to increase FGFR3 dimerization, but the dimerization propensities of wild-type and mutant FGFR3 have not been compared. Here we use quantitative imaging FRET to characterize the dimerization of wild-type FGFR3 and the ACH mutant in plasma membrane-derived vesicles from HEK293T cells. We demonstrate a small, but statistically significant increase in FGFR3 dimerization due to the ACH mutation. The data are consistent with the idea that the ACH mutation causes a structural change which affects both the stability and the activity of FGFR3 dimers in the absence of ligand.

## Introduction

Fibroblast growth factor receptor 3 (FGFR3) negatively regulates long bone growth by controlling the differentiation of chondrocytes in the growth plate [1]. Single amino acid mutations in FGFR3 are known to impact long bone development and to lead to pathologies [2]. Most of the known pathogenic mutations in FGFR3 are gain of function mutations which over-activate the receptor and cause premature chondrocyte differentiation. Thus, the proliferation stage for the chondrocytes is shortened due to the mutations, leading to a decrease in the overall length of the long bones [2]–[5].

One of the best known FGFR3 mutations is the Gly380Arg mutation in the transmembrane (TM) domain of the receptor [6]. This point mutation has been associated with 97% of the reported cases for achondroplasia (ACH), the most common form of human dwarfism [2], [7]. The ACH phenotype is characterized by short stature, bowed legs, and shortened arms and legs [8], [9]. The incidence rate of ACH is approximately one in 15,000 live births, and most of the cases are sporadic.

Since the discovery of the Gly380Arg mutation as the genetic cause for human dwarfism, research in the field has focused on the effect of this mutation on FGFR3 signaling. FGFR3 is a member of the receptor tyrosine kinase (RTK) superfamily. Thus, FGFR3 is a single pass receptor which consists of an extracellular ligand binding domain, a TM domain and an intracellular kinase domain, and functions via lateral dimerization in the membrane [10]–[12]. FGFR3 dimerization brings the two kinase domains in close proximity such that the two kinase domains can cross-phosphorylate and activate each other [13], [14]. This process is regulated by ligands from the *fgf* family, which bind to FGFR3 extracellular domain on the cell surface in the presence of heparin sulfates. The bound ligands are believed to stabilize the dimer, alter its structure and enhance its activation [15]–[18]. Thus, multiple physical interactions regulate FGFR3 activation, and a question arises as to which of these interactions is affected by the ACH mutation.

Published studies of the effect of the ACH mutation on FGFR3 signaling demonstrate that the mutation increases ligand-independent activation [17], [19]–[21]. However, the activation of FGFR3 at high ligand concentrations, and the binding of ligand (fgf1) to FGFR3, are not affected by the ACH mutation. Thus, the effect of the mutation is restricted to ligand-independent FGFR3 activation. The cause for this increase, however, is controversial. Webster and Donoghue hypothesized that the activity is increased because the mutation increases FGFR3 dimerization [19]. Their hypothesis was based on the observation that FGFR3 activity was increased, as compared to wild-type, when the glycine residue at position 380 was replaced with amino acids capable of forming hydrogen bonds. However, they did not compare the dimerization propensities of the wild-type and the mutant.

He *et al*. used cross-linking of the full-length FGFR3 in mammalian membranes to test the hypothesis that the ACH mutation increases FGFR3 dimerization [17]. Despite the increased FGFR3 activation at low ligand concentration due to the mutation, there was no discernible difference in the cross-linking propensities of the wild-type and the mutant. Instead, the ACH mutation was found to increase the probability for phosphorylation of tyrosines in the kinase activation loop, and was hypothesized to induce a structural change in the unliganded dimer [17].

A definitive conclusion about the effect of the mutation on dimerization cannot be drawn from this study [17], however, because cross-linking gels are difficult to quantify due to the non-specific nature of the cross-linker and because cross-linking propensities depend not only on dimerization, but also on structure. In particular, since the ACH mutation is believed to induce a structural change in the unliganded dimer [17], the cross-linking efficiencies for the wild-type and the mutant may be different. In this case, chemical cross-linking cannot be used as a reliable assay to compare dimerization. Thus, despite extensive research in the field, it is not yet known if the ACH mutation alters the dimerization propensity of FGFR3.

A rigorous test of the hypothesis that the ACH mutation increases FGFR3 dimerization requires an experimental methodology that yields dimerization constants and dimerization free energies for membrane proteins. While measurements of association constants are routinely performed for soluble proteins, the development of techniques that are applicable to membrane proteins is still in its infancy. Challenges arise because membrane proteins are difficult to overexpress and purify; yet, knowledge of exact protein concentrations is required for quantitative dimerization measurements [22]. For glycoproteins such as RTKs, non-mammalian expression systems are unsuitable, as they lack the appropriate post-translational modification machinery. We have shown, however, that all of these challenges can be overcome if measurements are carried out in vesicles from mammalian plasma membranes using a FRET-based method, Quantitative Imaging FRET (QI-FRET), which yields association constants for membrane proteins (and RTKs in particular) without the need for their purification [22], [23]. The RTKs are produced in mammalian cells, and thus they are post-translationally glycosylated prior to their delivery to the plasma membrane. Experiments are carried out in plasma membrane-derived vesicles, which bud off cells upon treatments that disrupt the cytoskeleton [24], [25]. The QI-FRET method yields the FRET efficiency *E*, as well as the concentration of donors and acceptors, *C_D_* and *C_A_*, in each plasma membrane-derived vesicle, and thus yields association constants and dimerization free energies [26].

We have used this method previously to demonstrate that the effect of FGFR3 extracellular domains on ligand-independent FGFR3 dimerization energetics is repulsive and on the order of 1 kcal/mole [27]. Here, we use the QI-FRET method to measure and compare the dimerization propensities of wild-type and mutant FGFR3 constructs in the plasma membrane of HEK293T cells, thus assaying directly the effect of the ACH mutation on FGFR3 dimerization. As the achondroplasia mutation affects ligand-independent FGFR3 signaling, here we focus on ligand-independent dimerization.

## Materials and Methods

### Plasmid constructs

The pRSET_mCherry plasmid was obtained from R. Tsien (University of California, San Diego) and pEYFP was obtained from M. Betenbaugh (Johns Hopkins University). Additionally, the template pcDNA3.1_FGFR3 was obtained from D. Donoghue (University of California, San Diego). For all plasmids used in this study, the pcDNA3.1(+) vector (Invitrogen) was utilized for expression in mammalian cells.

As described previously, the FGFR3 EC+TM construct consisted of the signal peptide, the extracellular and transmembrane domains, a flexible 15 amino acid linker (GGS)_5_, and either mCherry or eYFP at the C terminus [27]. The achondroplasia mutant, Gly380Arg, was created using a QuickChange II XL mutagenesis kit (Agilent Technologies) and the following forward and reverse primers with the point mutation (which occurs at nucleotide 1138) shown in bold:

(1)5′- GGC ATC CTC AGC TAC ***A***GG GTG GGC TTC TTC CTG-3′

(2)5′- CAG GAA GAA GCC CAC CC***T*** GTA GCT GAG GAT GCC-3′

### Cell culture, transfection and vesiculation

HEK 293T cells were a kind gift from Dr. M. Edidin, JHU. The cells were passed and frozen stocks were prepared. This cell line was used here and in experiments of FGFR3 activation described elsewhere [16], [28]. The cells were cultured in Dulbecco's modified Eagle medium supplemented with 10% fetal bovine serum (FBS, HyClone) and a final concentration of 4.5 g/L glucose. Six-well plates were seeded at a density of 2×10^5^ to 5×10^5^ cells per well and allowed to grow for 24 hours before transfection.

FuGENE HD (Roche) was used as a transfection reagent according to the manufacturer's protocol. Transfected cells were allowed to grow overnight. Prior to vesiculation, the cells were rinsed with phosphate-buffered saline (PBS) (pH 7.4) and incubated in serum free medium for 5 hours. The cells were then rinsed two to three times with phosphate-buffered saline containing 0.75 mM calcium chloride and 0.5 mM magnesium chloride (CM-PBS). After rinsing, the cells were incubated in 1 mL of vesiculation buffer (CM-PBS, with 25 mM formaldehyde and 0.5 mM 1,4-dithiothreitol) at 37°C for one hour. Glycine in PBS was added to the vesiculation buffer to a final concentration of 0.125 M in order to quench the formaldehyde. After vesicle production, the vesicles were transferred to chamber slides (Thermo Scientific, Nunc Lab-Tek II chambered coverglass) for image acquisition.

### Image acquisition and analysis

As reported previously [27], vesicles were imaged using a Nikon C1 scanning confocal microscope with a 60× water immersion objective. Three images (scans) were collected for each vesicle. The “donor scan” used a 488 nm excitation and collected intensity over the range of 500–530 nm. The “FRET scan” used a 488 nm excitation and collected intensity over the range of 565–615 nm. The “acceptor scan” used a 543 nm excitation and the intensity was collected with a 650 nm long pass filter. A Matlab^(R)^ program developed in the laboratory was used to process each vesicle and determine the intensity for each channel across the lipid bilayers as described [23].

From the acquired images of single vesicles and of standard solutions, we can directly calculate the concentration of acceptor (*C_A_*) from the acceptor scan intensity (*I_A_*):
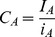
(1)The sensitized acceptor emission is determined by the following relationship:
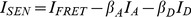
(2)where *ß_A_* and *ß_D_* are the bleed-through coefficients.

In order to determine the corrected (i.e., actual) donor intensity (*I_D,Corr_*), the calculated sensitized emission was multiplied by the Gauge factor *G_F_* (determined as described in [22]) and added to the observed donor channel intensity. This allows for the determination of the actual donor concentration from the observed donor scan intensity and the sensitized acceptor emission.
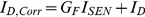
(3)

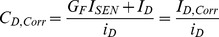
(4)From the corrected donor channel intensity, the FRET efficiency is determined as follows:
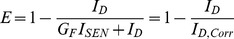
(5)In the next step, the FRET accounting for random proximity of donors and acceptors within distances of 100 Å or so was subtracted from the measured FRET efficiencies, yielding the actual FRET due to dimerization, (*E_D_*) [29]:
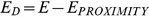
(6)The dimeric fraction (*f*) is then determined by the following relationship:
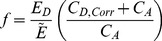
(7)where *E˜* is the FRET efficiency in a dimer with a donor and an acceptor [22].

The dimerization model used to fit the data is:
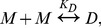
(8)where [*D*] and [*M*] and are the dimer and monomer concentrations. The dimerization constant is:
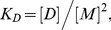
(9)and the total concentration [*T*] is given by:
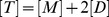
(10)The dimerization free energies are calculated from the dimerization constants *K_D_* according to equation (11):
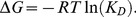
(11)


### Statistical Analysis

The dimeric fractions calculated for similar (within experimental error) total protein concentrations were grouped into bins of bin size 5.0×10^−4^ receptors/nm^2^. For each bin, the average dimeric fractions and the standard errors were calculated. Each bin contained between three and 43 data points corresponding to different single vesicles.

To determine if there is a difference between the averaged dimeric fractions for the wild-type and the mutant, a Chi Squared value was calculated for each bin according to:
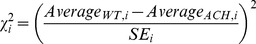
(12)The standard error for each bin, *SE_i_*, was calculated as:
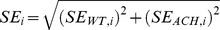
(13)In these equations, *Average_WT,i_* and *Average_ACH,i_* are the average dimeric fractions for the wild-type and the ACH constructs in each bin, *i*, with their respective standard error, *SE_WT,i_* and *SE_ACH,i_*.

The total reduced Chi Square for the whole curve was then calculated as:
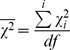
(14)where *df* is the number of degrees of freedom. The p-values for the calculated reduced Chi Squared values were determined using a Chi Squared table [30] with a p-value<0.05 considered significant.

## Results

### Dimerization measurements for wild-type and mutant FGFR3

Here, we directly measured the interactions of two different (wild-type and mutant) FGFR3 constructs consisting of: (i) the FGFR3 extracellular (EC) domain, (ii) the FGFR3 transmembrane (TM) domain, (iii) a 15 amino acid (GGS)_5_ linker and, (iv) a fluorescent protein, either mCherry or eYFP (a FRET pair). The plasmid design for the wild-type construct, which includes a cleavable signal sequence directing the receptor to the plasma membrane, is described in detail in [27]. This construct allows the implementation of the QI-FRET method and the calculation of dimerization free energies [23], [27], as full-length constructs express at very low levels. The (GGS)_5_ flexible linker is added to decouple the distance between the fluorescent proteins in the dimer (and thus, the FRET efficiency) from the structure of the dimer, yielding an assay that is sensitive to dimerization propensities but not structure [31]. The Gly380Arg mutation was created for this work by changing nucleotide 1138 from G to A in the transmembrane domain of the construct, as described in Materials and Methods.

The interactions were measured in plasma membrane derived vesicles produced from human embryonic kidney 293T (HEK293T) cells. This cell line has been previously used to study FGFR3 signaling [16], [28]. Cells were transfected with the genes encoding either the wild-type FGFR3 eYFP and mCherry constructs or the mutant FGFR3 eYFP and mCherry constructs. After overnight cell growth in standard media, the media was replaced with serum free media for five hours to ensure that no ligands were present [17], [28]. Cells were then vesiculated using a standard protocol [22], [23]. Briefly, the cells were rinsed with CM-PBS and then covered with a 1 mL solution of CM-PBS containing 25 mM formaldehyde and 0.5 mM DTT. Glycine was added in a twenty-fold excess to quench the formaldehyde as previously described [23]. After the completion of the vesiculation process, the vesicles were transferred to four-chambered slides and imaged.

**Figure 1:**
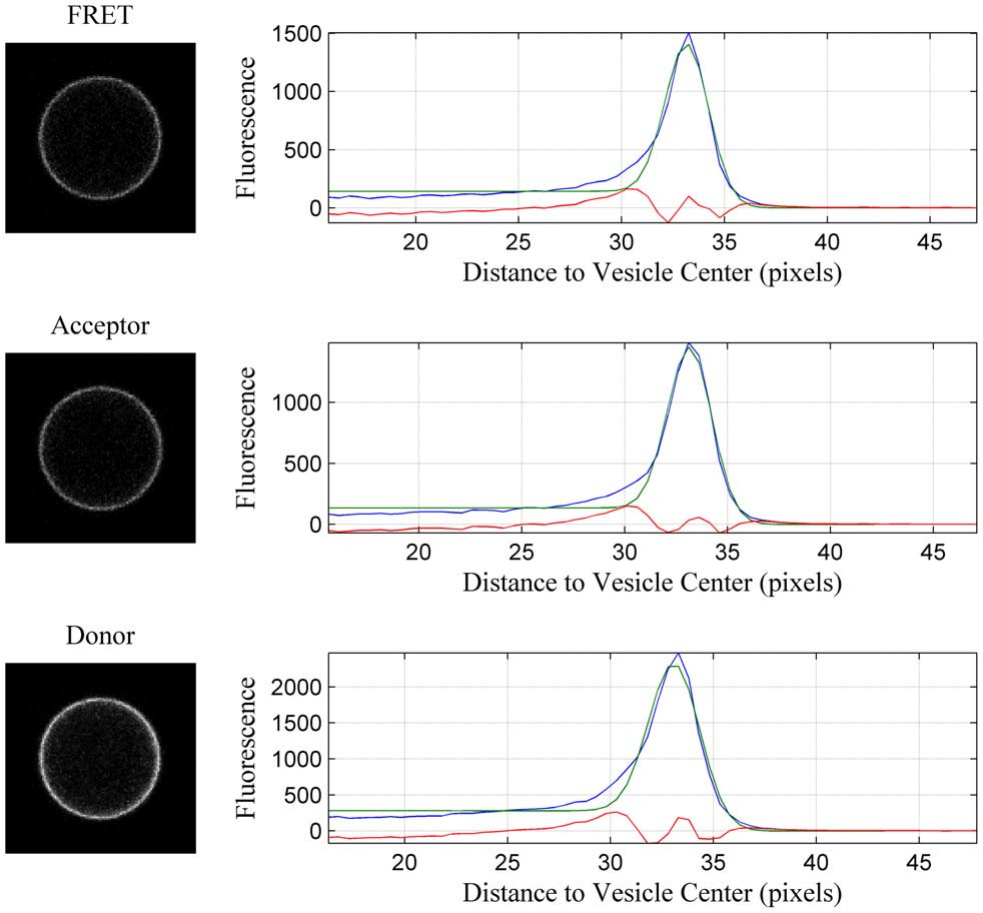
Donor, FRET and acceptor scans of a single vesicle. One vesicle co-expressing the two wild-type constructs tagged with eYFP (donor) and mCherry (acceptor) is imaged in three different scans: the donor (ex: 488 nm, em: 500–530 nm), FRET (ex:488 nm, em: 565–615 nm) and acceptor (ex: 543 nm, em: 650 nm long-pass) scans. The intensities across the membranes are obtained by summing along the vesicle circumference. They are shown as a function of the distance from the vesicle center (blue). After background correction, they are fitted with Gaussians (green) and the Gaussians are integrated to yield total channel intensities. The red curve is the difference between the data and the fit.

Images were acquired with a Nikon C1 laser scanning confocal microscope using a 60× water immersion objective as described in detail in [22], [23]. Three different scans were performed for each vesicle. In the donor scan the intensities were recorded over the range of 500–530 nm with an excitation source at 488 nm. The FRET scan recorded intensities over the range of 565–615 nm with an excitation of 488 nm. The acceptor scan measured intensities with a long pass filter (>650 nm) with an excitation source of 543 nm. The processed images from a single vesicle are shown in Figure 1. The vesicles were recognized by a Matlab code and the intensities across the bilayer were then determined for each channel [23]. The pixel intensities across the membrane were fitted with a Gaussian function, and the background fluorescence was modeled with an error function. Integration of the Gaussians yielded the three intensities: 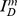 (from the donor scan), 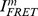 (from the FRET scan), and 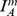 (from the acceptor scan), per unit membrane area [23]. As expected for membrane proteins, the fluorescence in Figure 1 is located on the membrane. Furthermore, the fluorescence is uniform, suggesting that the protein distribution in the vesicle is homogeneous, most likely due to the lack of the cytoskeleton which is known to maintain lateral membrane heterogeneity. As previously discussed, uniform fluorescence is critical for the calibration of the donor and acceptor concentrations in the vesicles and, in turn, the successful implementation of the QI-FRET method [22], [23].

In each experiment, protein standard solutions were also imaged as described in Li *et al* [22], with the goal of calibrating the intensity measurements and ultimately, determining the donor and acceptor concentration in each vesicle. Isolated and purified mCherry and eYFP solutions at four different concentrations (in the range 0.5 µM to 4 µM) were imaged in the confocal microscope using the donor, FRET, and acceptor scans. From these three scans, the coefficients relating intensities and concentrations for the donor and acceptor, *i_D_* and *i_A_,* were determined. The intensities of the purified protein solutions were linear with concentration and the slope of the experimental data yielded *i_D_* and *i_A_* [22], [23]. The bleed-through coefficients *ß_D_* and *ß_A_* were also measured for the purified donor and acceptor solutions. Finally, the Gauge factor, *G_F_*, relating the sensitized emission intensity to the donor quenching, was determined using a linked eYFP/mCherry construct as discussed in detail in [22].

**Figure 2:**
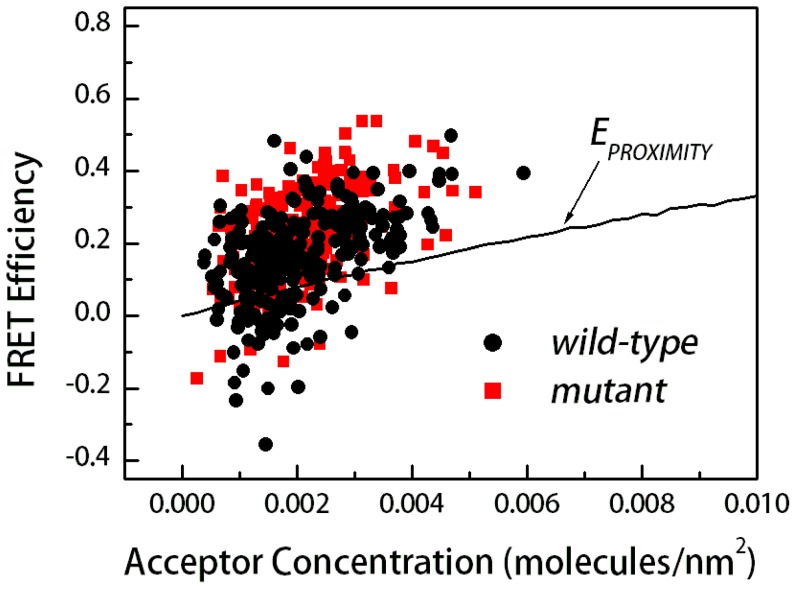
FRET data and proximity contribution for the wild-type and mutant constructs in HEK 293T plasma membrane-derived vesicles, as a function of acceptor concentration. Each data point represents a single vesicle, for which *E*, *C_A_* and *C_D_* are determined using the QI-FRET method. Black circles: FRET efficiencies measured for the wild-type construct (∼200 data points). Red squares: FRET efficiencies for the mutant construct (∼200 data points). Data scatter in this type of experiment is due to random noise in image acquisition, and is reducible by collecting a large number of data points [23].

From the acquired images of single vesicles and of standard solutions, the acceptor concentration (*C_A_*) was calculated from the acceptor scan intensity (*I_A_*) according to equation (1). The donor concentration was calculated using equation (4) and the FRET efficiency was given by equation (5). The FRET efficiency is shown as a function of acceptor concentration in Figure 2. Each data point in the figure corresponds to a single vesicle. We do not see obvious differences in the FRET efficiencies measured for the wild-type and the achondroplasia mutant. This result is consistent with previous findings that the cross-linking of the wild-type and mutant receptors is similar [17]. In the current study however, exact dimerization parameters can be computed and compared, as discussed below.

Since the membranes are two-dimensional structures, the attached fluorescent proteins are confined to move in two dimensions. Thus, we must account for FRET that occurs due to random (stochastic) proximity of the donors and acceptors (*E_PROXIMITY_*) within distances of 100 Å or so; we have done so in the past using the framework developed by Wolber and Hudson [32], while also taking into account the size of the fluorescent proteins [23]. This FRET contribution, shown in Figure 2 as a black line, will be measured even in the absence of specific interactions. We see that the measured FRET efficiencies, *E*, are greater than the predicted ones for proximity FRET, *E_PROXIMITY_*, indicative of specific interactions between FGFR3 molecules.

**Figure 3:**
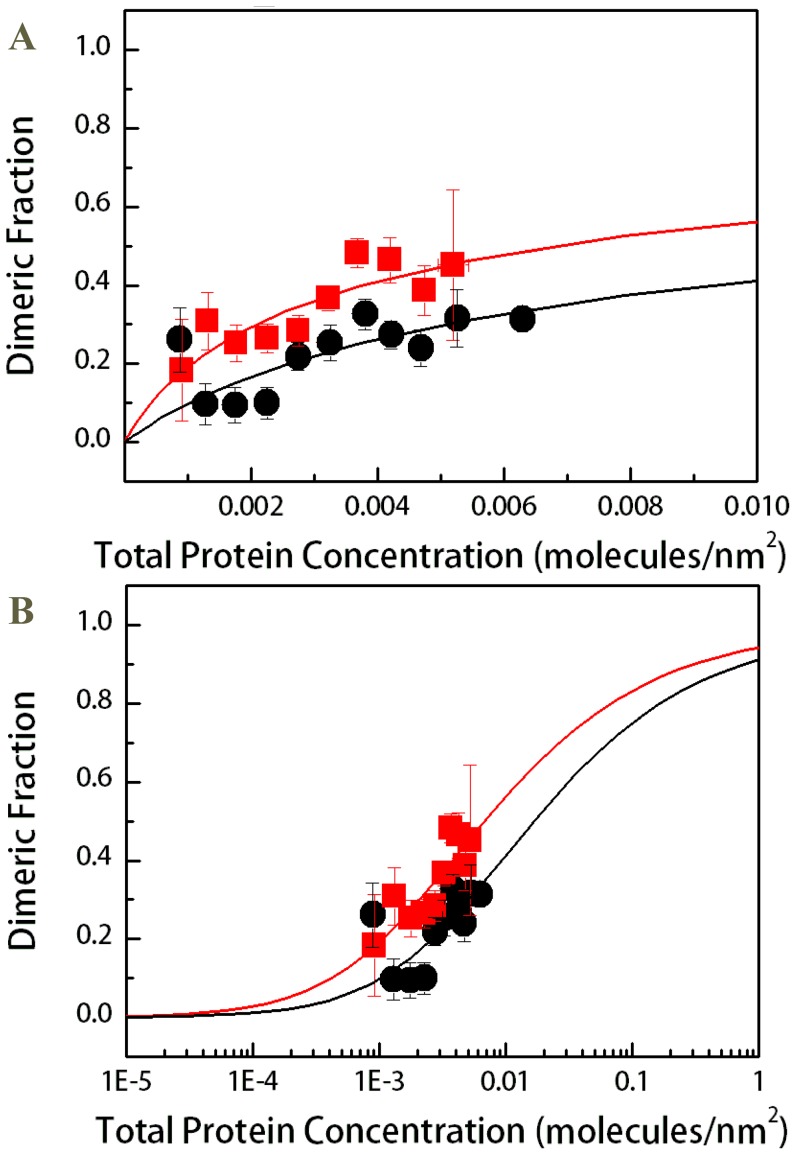
Binned dimeric fractions for the wild-type and mutant constructs as a function of total receptor concentration. (A). Dimerization curves. Black circles: Averaged dimeric fractions measured for the wild-type construct. Red squares: averaged dimeric fractions measured for the mutant construct. The data were fitted to the dimerization model given by equations (8)–(10), yielding the dimerization constants *K_D_*. The ACH mutation induces a statistically significant (p≪0.01) increase in dimer fraction. It stabilizes the FGFR3 dimer by ΔΔG = −0.5±0.3 kcal/mole. (B) All data are re-plotted on a semi-log plot.

The FRET due to proximity was subtracted from the measured FRET efficiencies, yielding the actual FRET due to dimerization (*E_D_*) (see equation 6). *E_D_* is needed to calculate the dimeric fractions according to equation (7). The parameter *E˜* in equation (7) is the FRET efficiency of a dimer with a donor and an acceptor [22]. The value of *E˜* is dependent upon the separation distance between the donor and the acceptor in the dimer, i.e. on the dimer structure. Here we used a flexible 15 amino acid linker to attach the fluorescent protein to the TM domain [33]; the same linker was used in previous studies of GpA dimerization [23]. *E˜* for this attachment was previously determined to be *E˜* = 0.63±0.04, corresponding to a 48.5 Å separation distance between the fluorophores in the dimer [23]. With *E˜* known, the dimeric fractions for wild-type and mutant FGFR3 were calculated from equation (7) for each vesicle. The dimeric fractions for vesicles with similar total receptor concentration were averaged within bins with bin size of 5×10^−4^ receptors/nm^2^. The binned results (dimeric fraction ± standard error versus total concentration ± standard deviation) are shown in Figure 3 (Figure 3B is a semi-log plot typically used to present binding data). These results demonstrate a small increase in the averaged dimeric fractions due to the mutation.

### Statistical analysis of the effect of the mutation on FGFR3 dimerization

The statistical significance of the increase in the measured dimeric fractions due to the mutation was determined using reduced Chi Squared analysis as discussed in Materials and Methods. Briefly, a reduced Chi Square value was calculated for each bin using equation (12). The total reduced Chi Square for the whole curve was then calculated using equation (14). The degrees of freedom *df* was equal to 9, the number of bins minus one. The reduced Chi Square value was calculated as 5.29, giving a p-value of less than 0.001. Thus, the increase in dimerization due to the achondroplasia mutation that we measure is statistically significant.

### Calculation of dimerization free energies

The dimeric fraction, *f* = 2[*D*]/[*T*], was calculated theoretically as a function of the total concentration [*T*] for various values of *K_D_*, based on the two-state dimerization model given by equations (8)–(10).

Using equations (8)–(10), we can predict the dimeric fraction *f* = 2[*D*]/[*T*] as a function of the total concentration [*T*] for any value of the dimerization constant *K_D_*. This prediction is compared to the experimentally determined single-vesicle dimeric fractions (prior to binning) while optimizing for the dimerization constant *K_D_* such that the predictions give the best fit to the experimental data. The optimal *K_D_* values for the two FGFR3 constructs in HEK 293T cells determined in the fit were 57.14 and 140.85 nm^2^ for the wild-type and the mutant, respectively. These optimal *K_D_* values were independent of the initial guesses used in the fit. The fits are shown in Figure 3 with the solid lines, allowing a direct comparison between the binned data and the fits.

The dimerization free energies of the wild-type and the mutant in HEK 293T cells were calculated from the dimerization constants *K_D_* according to equation (11). For the wild-type, Δ*G_WT_* was determined as −2.4±0.2 kcal/mol and for the mutant, Δ*G_ACH_* was −2.9±0.2 kcal/mol. The experimental errors in the dimerization free energies were calculated from the standard errors shown in Figure 3. Thus, the effect of the mutation on dimerization is ΔΔ*G* = −0.5±0.3 kcal/mole.

## Discussion

The TM domains of RTKs have been shown to play a critical role in stabilizing RTK dimers and in establishing the activation-competent dimer structure [34]–[36]. Mutations in RTK TM domains have been shown to interfere with the normal function of RTKs and cause disease [18], [37]. The ACH mutation, occurring in the TM domain of FGFR3, is one of the best known examples of a pathogenic RTK TM domain mutation.

It has long been accepted that the ACH mutation increases FGFR3 dimerization, which in turn increases FGFR3 activity and ultimately, leads to pathology [3], [38], [39]. While this view has been challenged [17], [21], [40], there have been no direct comparisons of the dimerization propensities of the wild-type and the mutant receptors. This lack of data has been due to a lack of methodology that yields dimerization propensities of complex membrane proteins in cellular membranes. We have developed a method, QI-FRET, that allows us to characterize the dimerization of complex glycosylated membrane proteins in mammalian membranes [23]. Here we used the method to characterize the dimerization of wild-type FGFR3 and the ACH mutant in plasma membrane-derived vesicles from HEK293T cells. We measured similar FRET efficiencies for the two constructs (Figure 2). Yet, by collecting a large number of data points and by performing statistical analysis, we demonstrated that the mutation increases FGFR3 dimerization in a statistically significant way (p≪0.01). However, the effect of the mutation is small, ΔΔ*G* = −0.5±0.3 kcal/mole. Such a small increase in dimerization has not been detectable in cross-linking experiments due to the inherent limitations of such experiments (see Introduction), and due to the fact that cross-linking is sensitive to both close contact and structure. In the FRET experiments, the dyes were attached to the receptor constructs using a flexible linker. Such a flexible linker has been shown to structurally decouple the proteins to which it is attached [31]. Thus, mutation-induced structural changes are not expected to affect the FRET efficiency.

Our previous investigations of the activation of FGFR3 in HEK293T cells have demonstrated a 2.5 fold increase in activation due to the mutation in the absence of ligand (but no effect in the presence of ligand) [17]. This increase, measured using quantitative Western blotting, is larger than the increase in dimerization measured here, while the expression levels are similar in the Western blot and FRET experiments. Thus, the increase in FGFR3 phosphorylation due to the ACH mutation cannot be attributed completely to the increase in dimerization propensity. Indeed, the efficiency for phosphorylation of critical Tyrosine residues in the kinase activation loop is increased due to the ACH mutation, most likely due to a mutation-induced structural change [17].

To some, previous reports that the ACH mutation affects the structure and the phosphorylation of FGFR3 unliganded dimers without affecting their stability [17] might have appeared counterintuitive. Dimerization propensities are expected to scale with the contact area between the two proteins in the dimer, and this contact area should ultimately depend on dimer structure. By using the QI-FRET method, here we demonstrate a small increase in dimer stability due to the mutation, consistent with the idea that the ACH mutation causes a structural change which affects both the stability and the activity of unliganded FGFR3 dimers. The structural change may also explain the reports of the compromised downregulation of the mutant receptors, as it likely impacts the recognition of the receptor by the endocytic machinery that is responsible for recycling or destruction of internalized receptors [41].

Perhaps the most interesting finding about the ACH mutation is that its effects on FGFR3 dimerization and activation are very modest. The modest effects observed may be the key to some observations that have been difficult to rationalize. For instance, while FGFR3 is expressed in all cell types, the achondroplasia phenotype is restricted primarily to the skeletal system. Furthermore, the ACH mutation has been associated with cancer, but there is no evidence for higher occurrence of cancer in achondroplasia patients [42]. Now that the physical-chemical consequences of the ACH mutation are established, measurements in different relevant human cell lines should delineate cell-specific biological effects that modulate FGFR3 signaling and contribute to the phenotype.
